# Randomized controlled trial of a coordinated care intervention to improve risk factor control after stroke or transient ischemic attack in the safety net: Secondary stroke prevention by Uniting Community and Chronic care model teams Early to End Disparities (SUCCEED)

**DOI:** 10.1186/s12883-017-0792-7

**Published:** 2017-02-06

**Authors:** Amytis Towfighi, Eric M. Cheng, Monica Ayala-Rivera, Heather McCreath, Nerses Sanossian, Tara Dutta, Bijal Mehta, Robert Bryg, Neal Rao, Shlee Song, Ali Razmara, Magaly Ramirez, Theresa Sivers-Teixeira, Jamie Tran, Elizabeth Mojarro-Huang, Ana Montoya, Marilyn Corrales, Beatrice Martinez, Phyllis Willis, Mireya Macias, Nancy Ibrahim, Shinyi Wu, Jeremy Wacksman, Hilary Haber, Adam Richards, Frances Barry, Valerie Hill, Brian Mittman, William Cunningham, Honghu Liu, David A. Ganz, Diane Factor, Barbara G. Vickrey

**Affiliations:** 1 Los Angeles County Department of Health Services, Los Angeles, California USA; 2 University of Southern California, Los Angeles, California USA; 3 Rancho Los Amigos National Rehabilitation Center, Downey, California USA; 4 University of California, Los Angeles (UCLA), Los Angeles, California USA; 5 Los Angeles County-University of Southern California (USC) Medical Center, Los Angeles, California USA; 6 Kaiser Permanente, Sacramento, California USA; 7 Harbor-UCLA Medical Center, Torrance, California USA; 8 Olive View-UCLA Medical Center, Sylmar, California USA; 9 Cedars Sinai Medical Center, Los Angeles, California USA; 10 Kaiser Permanente, Irvine, California USA; 11 Fielding School of Public Health, Department of Health Policy and Management, University of California, Los Angeles, Los Angeles, California USA; 12 Watts Labor Community Action Committee, Watts, California USA; 13 Worker Education and Resource Center, Los Angeles, California USA; 14 Esperanza Community Housing, Los Angeles, California USA; 15 School of Social Work, Edward R. Roybal Institute on Aging, and Daniel J. Epstein Department of Industrial and Systems Engineering, University of Southern California, Los Angeles, California USA; 16 RAND Corporation, Santa Monica, California USA; 17 Dimagi, Inc., Cambridge, Massachusetts USA; 18 Department of Veterans Affairs Greater Los Angeles Healthcare System, Los Angeles, California USA; 19 Icahn School of Medicine at Mount Sinai, New York, New York USA

**Keywords:** Community health worker, Stroke, Transient ischemic attack, Intracerebral hemorrhage, Vascular risk, Blood pressure, Coordinated care, Disparities, NINDS Common Data Elements, Biomarkers

## Abstract

**Background:**

Recurrent strokes are preventable through awareness and control of risk factors such as hypertension, and through lifestyle changes such as healthier diets, greater physical activity, and smoking cessation. However, vascular risk factor control is frequently poor among stroke survivors, particularly among socio-economically disadvantaged blacks, Latinos and other people of color. The Chronic Care Model (CCM) is an effective framework for multi-component interventions aimed at improving care processes and outcomes for individuals with chronic disease. In addition, community health workers (CHWs) have played an integral role in reducing health disparities; however, their effectiveness in reducing vascular risk among stroke survivors remains unknown. Our objectives are to develop, test, and assess the economic value of a CCM-based intervention using an Advanced Practice Clinician (APC)-CHW team to improve risk factor control after stroke in an under-resourced, racially/ethnically diverse population.

**Methods/design:**

In this single-blind randomized controlled trial, 516 adults (≥40 years) with an ischemic stroke, transient ischemic attack or intracerebral hemorrhage within the prior 90 days are being enrolled at five sites within the Los Angeles County safety-net setting and randomized 1:1 to intervention vs usual care. Participants are excluded if they do not speak English, Spanish, Cantonese, Mandarin, or Korean or if they are unable to consent. The intervention includes a minimum of three clinic visits in the healthcare setting, three home visits, and Chronic Disease Self-Management Program group workshops in community venues. The primary outcome is blood pressure (BP) control (systolic BP <130 mmHg) at 1 year. Secondary outcomes include: (1) mean change in systolic BP; (2) control of other vascular risk factors including lipids and hemoglobin A1c, (3) inflammation (C reactive protein [CRP]), (4) medication adherence, (5) lifestyle factors (smoking, diet, and physical activity), (6) estimated relative reduction in risk for recurrent stroke or myocardial infarction (MI), and (7) cost-effectiveness of the intervention versus usual care.

**Discussion:**

If this multi-component interdisciplinary intervention is shown to be effective in improving risk factor control after stroke, it may serve as a model that can be used internationally to reduce race/ethnic and socioeconomic disparities in stroke in resource-constrained settings.

**Trial registration:**

ClinicalTrials.gov Identifier NCT01763203.

## Background

### Background and rationale

A small group of individuals with multiple comorbidities account for a disproportionate share of health care costs; in the United States, 10% of patients account for 70% of total health care expenditures [1]. These individuals have multiple chronic conditions, frequent hospitalizations, and often have limited ability to perform basic daily functions due to physical, mental, or psychosocial challenges. In recent years, coordinated care management models targeting these high-cost, high-need patients have been proposed to improve quality, reduce disparities, and minimize unnecessary healthcare spending [2–5].

Individuals with stroke or transient ischemic attack (TIA) are an important example of those high utilizers given their complex needs, including disability, multiple medical comorbidities, and highly prevalent concomitant depression. They have high rates of recurrent strokes and other future cardiovascular events. In fact, a prior stroke or TIA is the strongest predictor of a subsequent stroke, and the cumulative 5-year risk of recurrent stroke ranges from 15 to 25% [6]. Furthermore, the overwhelming majority of strokes each year could be prevented through awareness and optimal management of hypertension, and through lifestyle changes to healthier diets, greater physical activity, and smoking cessation [7, 8]. These four factors plus abdominal obesity account for 82 and 90% of the population-attributable risk for ischemic stroke and for hemorrhagic stroke, respectively [9]. Hypertension is the most important modifiable risk factor for stroke worldwide and is responsible for approximately two-thirds of cerebrovascular disease burden [10]. Although recurrent stroke risk can be substantially reduced by controlling modifiable vascular risk factors, these risk factors are optimally controlled in only a small proportion of stroke survivors [11–13]. Under-resourced blacks and Latinos in particular, have well-documented and substantial disparities in risk factor control as well as quality of care, and outcomes after stroke [14, 15].

To redress disparities in risk factor control after stroke or TIA, it is critical to enhance access to high quality health care services and to use patient-centered, culturally- and individually-tailored methods to improve lifestyle habits, health literacy, self-management skills, and medication adherence. The Chronic Care Model (CCM), advocated as a guide for developing care improvement interventions for patients with chronic disease, has been an effective framework for multi-component intervention programs aimed at improving care processes and outcomes while reducing costs for various chronic conditions, including diabetes and hypertension [16, 17]. The six components of this model are self-management support, clinical information systems, delivery system redesign, decision support for application of evidence-based care guidelines, health care organization championship and leadership support, and community resources. We recently completed a different randomized controlled trial (RCT) of a CCM-based intervention for secondary stroke prevention, Systemic Use of STroke Averting INterventions (SUSTAIN) [18]. This experience highlighted our need for a strong community-based component to intensively address behavioral risk factors by accounting for social determinants of health, such as the social and community context, living situation, physical environment, education, and access to healthcare.

CHWs – trained lay members of the community who serve as patient and community advocates, or “coaches” for disease management– have been engaged in the healthcare workforce for decades in low-income countries. CHWs are now also receiving heightened attention in high-income countries as healthcare is evolving to recognize and reimburse for a broader range of potential providers than physicians, physician assistants, and nurse practitioners, and to incorporate cultural competency in interventions targeting disparities in health. CHW interventions have been successful in various US populations with chronic conditions such as hypertension and diabetes, demonstrating significant improvements in health literacy, risk factor control, self-management skills, lifestyle habits, and a decrease in inappropriate health care utilization [19–27]. Few studies have evaluated the effectiveness of CHW interventions among stroke survivors, [28, 29] but none have evaluated such interventions in conjunction with a broader CCM-based healthcare system re-design.

### Objectives

**Fig. 1:**
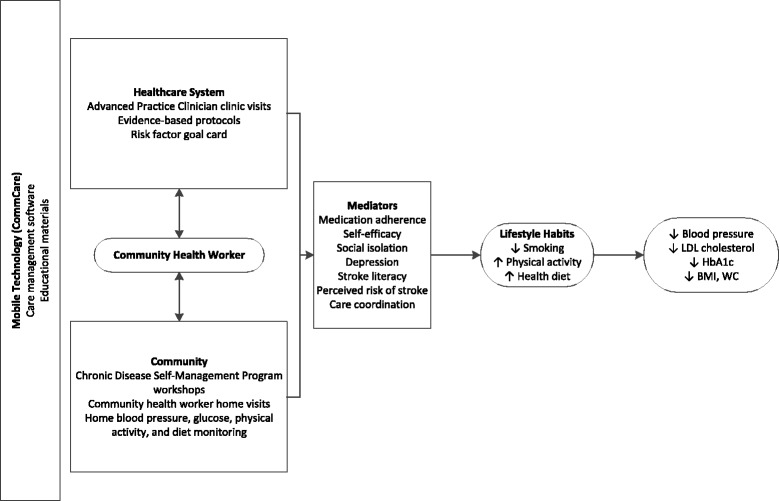
Conceptual model of SUCCEED intervention

Our objectives are to develop, test, and evaluate the effectiveness and assess the economic value of CCM-based intervention, Secondary stroke prevention by Uniting Community and Chronic care model teams Early to End Disparities (SUCCEED), to improve risk factor control after stroke in an under-resourced, racially and ethnically diverse population. The intervention consists of care from a team consisting of an Advanced Practice Clinician (APC) who is either a Nurse Practitioner (NP) or Physician Assistant (PA) and a CHW providing care in the healthcare setting (via clinic visits) and the community (through home visits and self-management group workshops held in community venues). The APCs and CHWs use mobile technology to communicate with each other, manage patient panels, reinforce self-management skills, employ culturally-, language-, and education level-appropriate patient education tools, and follow protocols of care, with decision support. We hypothesize that by promoting health care access, evidence-based care, care coordination, health literacy, self-management skills, a healthy lifestyle, and medication adherence; recognizing and treating depression, and minimizing social isolation, we can improve vascular risk factor control - particularly blood pressure (BP) - among a vulnerable population of stroke survivors (Fig. 1).

### Trial design

SUCCEED is an RCT, randomizing participants in a 1:1 fashion to usual care vs. intervention, stratified by site, language (English, Spanish, or other [Mandarin/Cantonese and Korean]), and type of stroke (ischemic vs. hemorrhagic).

## Methods: participants, interventions, and outcomes

### Study setting

Patients are enrolled from the four safety-net public Los Angeles County-Department of Health Services (LAC-DHS) medical centers and one private medical center that serves the indigent. The four hospitals that anchor care for patients in the LAC-DHS system are Los Angeles County-University of Southern California Medical Center, Harbor-UCLA Medical Center, Olive View Medical Center, and Rancho Los Amigos National Rehabilitation Hospital (composed of an acute care hospital and an acute rehabilitation hospital). This system serves the largest, most ethnically diverse county in the United States. LAC-DHS provides healthcare to 700,000 people each year and treats more than 300,000 emergency and trauma victims annually. Through an integrated network of hospitals, health centers and clinics, the LAC-DHS system makes medical and preventive care services accessible in communities across the county. Over 90% of individuals who utilize this system are from socioeconomically disadvantaged minority groups and the majority are Latino. A retrospective review of all stroke/TIA admissions from 2007 to 2012 at Rancho Los Amigos revealed the following racial/ethnic distribution: 58% Latino, 20% Asian, 13% non-Latino black, 7% non-Latino white, and 2% Native American/other. Only 3% had private insurance and 10% had Medicare; the remainder were uninsured or had Medicaid insurance [30]. According to Los Angeles County administrative databases, less than 50% speak English at home. The fifth site is Cedars-Sinai Medical Center; we limited enrollment to individuals residing in the Centinela Valley, an underserved area of Los Angeles County.

### Eligibility criteria

Inclusion criteria include (1) TIA, ischemic stroke or intracerebral hemorrhage within the prior 90 days and (2) either systolic blood pressure (SBP) ≥130 mmHg, or SBP between 120 and 130 mmHg and a history of hypertension or on antihypertensive medications prior to the stroke or TIA. Exclusion criteria are: (1) younger than 40 years old; (2) unable to communicate understanding of the study during the informed consent process, or (3) not fluent in English, Spanish, Korean, Mandarin or Cantonese. Individuals younger than 40 years of age are excluded because the mechanisms for their strokes are often not due to atherosclerosis (e.g. arterial dissections, inherited coagulation defects, cardiac conditions); therefore, the interventions in this trial may not target their risk factors. The included languages reflect the languages spoken by the majority of the LAC-DHS safety net population.

### Intervention structure and activities

**Fig. 2:**
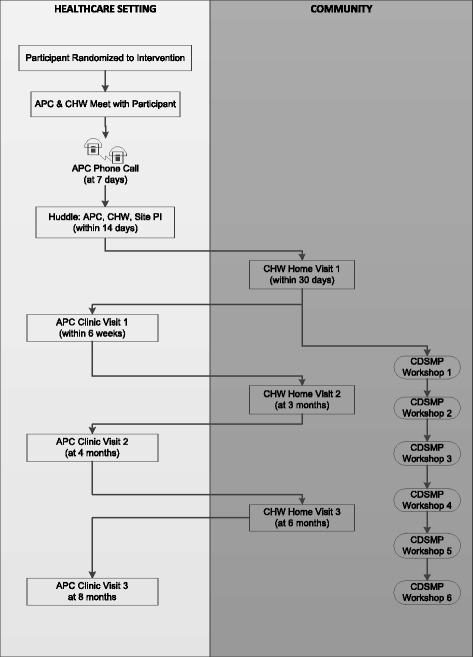
Participant flow in intervention

Individuals randomized to the intervention arm receive usual care plus care from a team consisting of an APC, supported by the site principal investigator (who is a Vascular Neurologist or Cardiologist), and a CHW. Over the course of the year, the intervention consists of: (1) a minimum of three clinic visits with the APC (CHW attends when possible); (2) a minimum of three home visits by the CHW; (3) the option to participate in Chronic Disease Self-Management Program (CDSMP) workshops, and (4) telephone coordination of care (Fig. 2). Beyond the minimum set of interactions, the care team has the ability to deliver additional interactions based on participants’ needs; therefore, individuals with more complex needs are tagged as “high needs” and receive more ‘touches’ than those who are more stable. Criteria that flag an intervention arm participant as “high needs” are: (1) SBP > 180 mmHg; (2) HbA1c > 10% or fingerstick glucose >200 mg/dL in the last 2 weeks; (3) depression (Patient Health Questionnaire [PHQ]-9 score > 10) or suicidal ideation, (4) morbid obesity (body mass index [BMI] > 35 kg/m^2^; (5) cognitive deficits impairing capability for self-management, and (6) alcohol or drug abuse. This is a fluid designation, and all participants are evaluated by the care team each week for their “needs status”. Participants have the choice to opt out of any aspect of the intervention. Intervention teams and the two study PIs have weekly conference calls to monitor and troubleshoot intervention implementation.

#### Care ManagementTechnology (CMT)

To increase effectiveness and efficiency, we contracted with Dimagi, whose platform, CommCare, is an open-source, mobile device-based tool for CHWs. The platform consists of two components - a mobile application, CommCare Mobile, and a cloud-based application management and reporting tool, CommCareHQ.

**Fig. 3:**
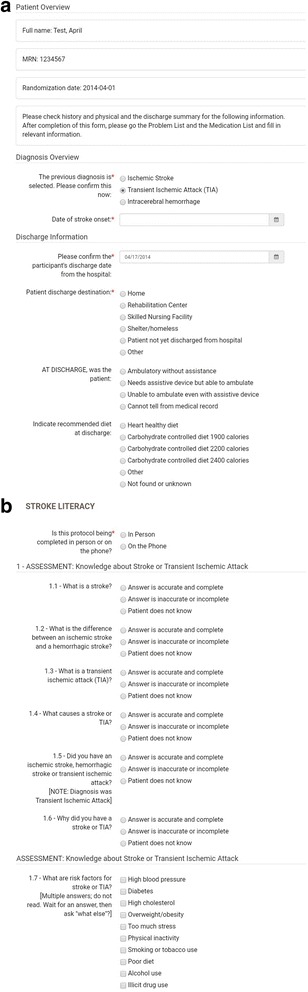
**a** Screenshot of CommCare application for APC. **b** Screenshot of CommCare application for CHW

The goal of CommCare Mobile was to enhance the CHWs’ capabilities during home visits. We programmed all protocols into CommCare Mobile, which was accessible via Android tablets (Fig. 3). CommCare Mobile was intended to enable the CHWs to: have immediate access to patient-specific care management information, including problem lists, care plans, risk factor goals, and health status; follow protocols, with decision support; administer depression, self-management, and lifestyle habit assessments; review participants’ goals; track tasks, complete and record assessments, and communicate with the APCs, and select culturally-tailored educational materials, including handouts, self-management tools, and videos. Data entered into CommCare Mobile is stored on the mobile device until a cellular connection is established to perform a two-way synchronization to CommCareHQ. CommCare HQ has reporting tools to provide real-time updates and can export data into other common software programs. Prior to this RCT, the platform had not been adapted to the specific topic of vascular risk factor management.

The APCs have the choice of using CommCareHQ on a desktop or CommCare Mobile on a tablet or smart phone. Updated data on any patient or task is passed back and forth between the CHWs’ and APCs’ tools. We pilot-tested the CMT with the CHWs and APCs using simulation data. Tracking tools installed on the CommCare platform enable the study team and the intervention teams to track forms submitted on a weekly basis.

#### CommCare formative evaluation

During the first 12 months of rollout of the CMT, we conducted a formal formative evaluation, to enable APCs and CHWs to provide feedback to revise the tools and protocols for more effective and efficient operation [31]. Overall, the APCs and CHWs expressed that the CMT assisted them in care management. The CMT helped APCs and CHWs track their interactions with participants and plan their work accordingly to ensure that participants received at least the minimum interactions per the SUCCEED protocol. For CHWs, the CMT facilitated efficient data collection, provided decision support during home visits, tracked participants’ stroke risk factor values, guided them in developing care plans, and provided remote access to care plans and important patient health information while in the community.

Nonetheless, the CMs and CHWs experienced numerous challenges when using the CMT. At the platform level, the most salient challenges were long loading times, inconsistent service reliability, and inaccurate information on reports due to the CMT not registering submitted forms. Major usability issues included difficulty navigating lengthy forms, the effort to manage tasks, and the inability of users to tailor reports, display educational materials, and obtain useful visualizations of stroke risk factor values. Finally, issues with the design of the content—extensive use of free-text responses and manual duplication of data that was already contained in the hospital’s database—greatly hindered care team efficiency. We worked with DiMagi to iteratively improve the CMT to meet the team’s needs.

#### Team communication

The care team (APC/CHW/PI) at each site holds weekly “huddles” to review new participants and jointly develop treatment plans, communicate about participants’ progress, and address problems. The CHW and APC meet more frequently to discuss participants in detail and determine participants’ “needs status”.

#### APC roles and activities

The APC roles include teaching self-management skills, such as BP and glucose monitoring, prescribing and titrating medications, and emphasizing medication adherence. The APC sees participants in clinic and coordinates care via telephone.

#### APC phone call

Within 1 week of enrollment in the intervention, the APC calls the participant and assesses current health status (any changes in stroke symptoms since enrollment), blood pressure, medication adherence and side effects, smoking, transportation, and reminds him/her of the next clinic appointment.

#### APC clinic visits

At clinic visits, the APC is guided by evidence-based care protocols developed by the research team, covering: stroke literacy, blood pressure, cholesterol, diabetes, antithrombotic medications, smoking cessation, depression, diet, and physical activity. Each protocol is organized into six sections: (1) assessment; (2) information provision; (3) self-management and adherence; (4) adjustment of medications (if applicable); (5) clinical support (e.g. from the site PI), and (6) resource provision for the participant. Clinic visits are conducted using patient-centered principles of care that include the patient’s chosen support network (caregivers, family, and friends). The APCs have smart cellular telephones that enable the participants to reach the APC during business hours by phone call or text message, as well as send photos of BP and glucose logs or new medications. The APCs also assist the participants with goal setting.

#### CHW roles

The CHWs key roles are to: (1) reinforce and enhance self-management skills; (2) serve as a liaison between the patient and the health care system; (3) mobilize resources, system support, and friend and family networks to reduce social isolation; and (4) educate participants about vascular risk factors, signs and symptoms of stroke, and activation of emergency medical services for stroke symptoms. These functions are performed through two mechanisms: CDSMP workshops held in the community, and home visits. The CHWs also have cellular telephones enabling participants to call the CHWs during business hours with questions and concerns. The CHWs assist the participants with transportation arrangements.

#### CHW home visits

The CHWs follow protocols to assess and address: (1) stroke literacy; (2) BP; (3) antithrombotic use; (4) cholesterol; (5) diabetes; (6) psychosocial issues, including depression and social isolation; (7) diet; (8) physical activity; (9) smoking; (10) alcohol and illicit drug use; (11) transportation, (12) communication preferences, and (13) access to care. The CHWs provide the participants with appropriate literacy-level, culturally-adapted educational materials (for Hispanic, African American, Chinese and Korean racial/ethnic groups) that were developed in conjunction with community-academic teams.

#### Chronic disease self-management program (CDSMP) workshops

Each workshop series is facilitated by two lead CHWs. Each series is comprised of 6 weekly workshops addressing key themes: use of symptom management techniques to deal with chronic disease symptoms, including fear, depression, anger, frustration, fatigue, pain and isolation; appropriate exercise for maintaining and improving strength and endurance; appropriate use of medications; communicating effectively with family, friends and health care professionals; and nutrition. Each session is framed by action planning, disease-related problem solving, and decision making. Trained CDSMP facilitators follow a scripted Leaders’ Manual each time they lead the program. Since the workshops are highly participatory, they work best when attended by at least seven participants. In order to increase the likelihood of meeting the seven minimum participants, intervention subjects are encouraged to invite caregivers, friends, family members, and community members with chronic conditions ranging from hypertension to obesity to participate. If the attendance is below seven, our community-based partners assist us in identifying participants for the workshop and we invite individuals not enrolled in the RCT to participate by advertising the workshops in outpatient clinics. Each intervention subject in the workshop receives a personal copy of the companion book, *Living a Healthy Life with Chronic Conditions, 3rd Edition* and an audio relaxation CD, *Time for Healing*. Participants are provided with small incentives for participation in the CDSMP workshops, such as $25 gift cards for grocery stores and an alarm/CD player to play the CDSMP meditation CD. Attendance at CDSMP workshops is recorded in CommCare.

The CHWs received 9 days of training to lead CDSMP workshops. In order to address cultural differences, the trainings for English and Spanish were separate. Two CHWs facilitate CDSMP workshops; separate workshops are conducted for English and Spanish-speaking intervention participants.

#### Education and support materials

In addition to the materials described above, other materials are provided to support the decision-making and behavioral choices of participants.

#### Goal cards and tools

**Fig. 4:**
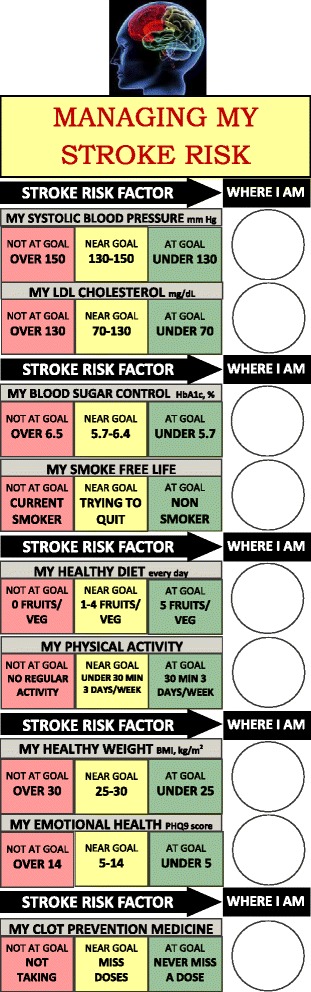
Risk factor goal card: trifold wallet card

At the first clinic visit with the APC, participants receive a customized goal card delineating current versus optimal control of key factors: BP, low density lipoprotein (LDL) cholesterol, diabetes, smoking, diet, physical activity, BMI, emotional health, and antithrombotic medications (Fig. 4). Factors are classified as “at goal” (green), “near goal” (yellow), and “not at goal” (red). Participants have the option of choosing media with this information; choices include refrigerator magnets, whiteboards, accordion pocket cards, coffee mugs, water bottles, and spiral bound index cards. Some of the goal tools, such as the white board, include a space labeled “where I am” and space to write action plans. Goal tools were developed using input and feedback from approximately 10 individuals attending a Stroke Community Engagement Symposium, a local event dedicated to raising awareness and arming participants with information on stroke warning signs, risk factors, treatment, and disparities. Attendees included stroke survivors, community advocates, and members of community-based organizations. Further input on the design, content, language, practicality, cultural sensitivity, and usefulness of the goal cards was obtained from 15 stroke survivors recruited from Rancho Los Amigos.

#### Blood pressure self-management and glucose monitoring

Once participants are randomized to the intervention, they receive Omron HEM-711 DLX BP monitors for home monitoring and are instructed on how to use the BP monitor and log and track the results. Participants also receive instructions on how to check and monitor blood glucose levels, if clinically indicated. These skills are assessed and re-enforced at subsequent clinic visits and home visits.

#### Intervention team recruitment and training

The PIs and site PIs trained the APCs to follow evidence-based protocols, teach self-management skills, and educate participants. Dimagi conducted remote and on-site training to use CommCare Mobile and CommCare HQ. The APCs also received training on motivational interviewing.

We partnered with three community-based organizations to recruit CHWs: (1) the Los Angeles Healthcare Workforce Development Program (HCWDP)/Worker Education and Resource Center (WERC), (2) Esperanza Community Housing Corporation (Esperanza), and (3) Watts Labor Community Action Committee (WLCAC). HCWDP is a labor/management partnership between LAC-DHS and the Service Employees International Union, that provides workforce development courses for healthcare workers, and specifically for CHWs who work in the Los Angeles healthcare safety-net system of hospitals and clinics. Esperanza and WLCAC are nonprofit organizations empowering residents of South Central Los Angeles through programs focused on affordable housing, health, arts and sciences, education, and economic development. To date, Esperanza’s Community Health Promoters Training Program has trained over 400 bi- and trilingual low-income residents to become community health leaders, patient advocates, health educators, and community organizers.

We conducted CHW training and selection in two phases. First, Esperanza, WLCAC, and WERC advertised the opportunity to obtain training in CDSMP and stroke to their community members and CHWs. We selected bilingual (English and Spanish) individuals to complete a 36-h training workshop for CDSMP. Second, we invited graduates of the CDSMP training to participate in an 80-h training on SUCCEED-specific topics, including stroke and vascular risk factors. We selected CHWs from the second phase of training.

We developed the 80-h curriculum for CHWs in collaboration with WERC. Topics included basic CHW skills, stroke etiology, signs and symptoms of stroke, hypertension, obesity, diabetes, dyslipidemia, diet, physical activity, smoking cessation, communication, speech, and swallowing, mental health and cognition, self-management skills, motivational interviewing, assessing therapy needs, potential stroke complications, common medications for stroke, tools for medication adherence, healthcare system navigation, goal cards and tools, panel management, and staff safety. The courses were taught by study staff, subject matter experts, and WERC curriculum developers and instructors. We trained two cohorts of CHWs—one in 2013 and another in 2015 (due to attrition of one CHW during the trial and expansion of the trial to the private medical center).

### Control structure and activities

Participants randomized to the control arm receive usual care, which varies by study site, but typically includes primary care visits, a post-hospital follow up visit with a vascular neurologist, and AHA educational handouts regarding stroke, physical activity and diet, and antithrombotics.

### Clinical outcomes

The primary outcome is the proportion of participants who achieve BP control (SBP <130 mmHg) at 1 year and the average change in SBP (Table 1. Secondary outcomes include: (1) mean change in systolic BP; (2) control of other vascular risk factors including lipids and hemoglobin A1c, (3) inflammation (C reactive protein [CRP]), (4) medication adherence, (5) lifestyle factors (smoking, diet, and physical activity), (6) estimated relative reduction in risk for recurrent stroke or MI, and (7) cost-effectiveness of the intervention versus usual care (Table 1). In addition, we will estimate the combined impact of changes in multiple risk factors on the relative risk of cardiovascular disease (CVD) or recurrent stroke in intervention and control groups. This secondary outcome of “global CVD risk reduction” will be modeled using observed changes in risk factor values for each participant and the relative risk reductions reported in randomized controlled trials of respective interventions (ie: statin therapy, anti-hypertensive medications). Detailed methods used to calculate the global risk reduction will be published in a forthcoming manuscript.

**Table 1:** Primary and secondary outcomes, moderators, and mediators

Outcome	Measure
Primary Outcome	
Systolic blood pressure control (<130 mmHg)	Physical exam
Secondary Outcomes	
Change in systolic blood pressure	Physical exam
Dyslipidemia: non-HDL cholesterol	CardioChek®
Glucose control: hemoglobin A1c	Dried blood spot
Inflammation: C-reactive protein	Dried blood spot
Adiposity: BMI, WC, WHR	Physical exam
Physical activity	Adapted from International Physical Activity Questionnaire [59]
Diet	California Health Interview Survey (CHIS) 2011–2012 [60]
Smoking	CHIS 2011–2012, Behavioral Risk Factor Surveillance System (BRFSS) 2013 [61]
Recurrent stroke or TIA	Questionnaire for Verifying Stroke-Free Status (QVSFS) [62]
Myocardial infarction	Medical history Common Data Element [45]
Cost	
Moderators	
Sociodemographics: age, sex, race/ethnicity	NINDS Demographics Common Data Element [45]
Acculturation and Education: country of birth, primary language, education level	Adapted from NINDS Demographics CDE, subscale of Bidimensional Acculturation Scale for Hispanics [63]
Health-care system	Study site
Type of cerebrovascular event (TIA, ischemic stroke, intracerebral hemorrhage)	Medical record
Stroke severity	NIH Stroke Scale
Functional status	Modified Rankin Scale
Mediators	
Stroke Literacy	Stroke warning signs and risk factors for stroke [64]
Health Literacy	4-Item Brief Health Literacy Screening Tool (BRIEF) [65]
Medication adherence	Adapted from Simoni et al. [66] and Chesney et al. [67]
Self-management skills	Adapted from Self-Efficacy for Managing Chronic Disease 6-item scale [68]
Self-efficacy	General Self-Efficacy Scale [69]
Perceived risk of stroke	Adapted from Stroke Risk and Worry Survey [70]
Social isolation	8-Item Social Support Scale [71]
Depression	Center for Epidemiologic Studies Depression Scale (CES-D) [72]
Health related quality of life	SF-6D [73]
Perceptions of quality of care	Adapted from Patient Assessment of Chronic Illness Care (PACIC) [74] and Consumer Assessment of Healthcare Providers and Systems (CAHPS®) [75]
Intervention Mediators	
Coordination and communication with care team: number CHW home visits, CDSMP workshops attended, APC clinic visits, telephone visits; communication between APC and CHW	CommCare tracking technology

*Abbreviations*: *BMI* body mass index, *WC* waist circumference, *WHR* waist-to-hip ratio, *CDE* common data element

### Participant timeline

**Fig. 5:**
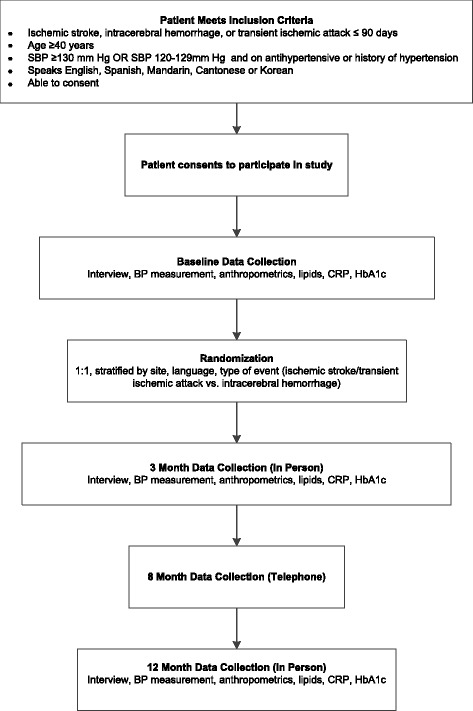
Enrollment of subjects and schedule for collecting evaluation data

At baseline, 3 and 12 months, research assistants (RAs) interview participants and measure BP, anthropometrics (including height, weight and waist circumference), and laboratory studies (by fingerstick, using a CardioChek® handheld device and dried blood spot collection; Table 2, Fig. 5). After the participant sits still for five minutes, three BP measurements are obtained five minutes apart; the mean BP is used. Participants complete a telephone-administered interview at 8 months. Subsequently, after the 12-month study period, participants receive surveillance telephone calls every 6 months to assess for vascular events or death up to 36 months. Weekly reports of screening, enrollment, and data collection by time point and by site are generated from Research Electronic Data Capture (REDCap™) and circulated and discussed on weekly investigator/evaluation RA conference calls.

**Table 2:** Evaluation timeline

		“On” intervention or usual care			“Off” or post-intervention or usual care follow up		
Time, months	Baseline	3	8	12	18	24	30
Informed consent	X						
Physical exam ● Blood pressure ● Body mass index ● Waist circumference ● Waist-to-hip ratio ● NIH SS ● mRS	X	X		X			
Fingerstick labs ● Total cholesterol ● HDL ● Triglycerides ● Calculated LDL ● Total cholesterol/HDL ● Hemoglobin A1c ● C-reactive protein	X	X		X			
Full in-person^a^ questionnaire	X	X		X			
Brief telephone questionnaire			X				
Telephone surveillance (vascular events, death)					X	X	X

*Abbreviations*: *NIH SS* National Institutes of Health Stroke Scale, *mRS* modified Rankin Score, *HDL* high density lipoprotein cholesterol, *LDL* low density lipoprotein cholesterol

^a^Sometimes obtained by phone if an in-person visit was infeasible

### Sample size calculation and power analysis

Our sample size calculation and power analyses were based on the primary outcome of SBP. Meta-analyses have revealed a 25 to 40% reduction in recurrent stroke risk with BP-lowering therapies [32–34]. We initially defined BP control as SBP < 120 mmHg, consistent with the Seventh Report of the Joint National Committee on Prevention, Detection, Evaluation, and Treatment of High Blood Pressure (JNC-7). During the course of the trial, evidence of a potential J-shaped curve emerged among individuals with existing cardiovascular disease or diabetes, with higher cardiovascular events and mortality associated with tight BP control [35–38]. Retrospective analyses of RCTs and observational studies suggested a potential J-shaped curve among individuals with stroke as well, particularly in the first 6 months post-stroke [39–42]. Sixteen months after initiating the trial (after enrolling 150 participants), we increased our SBP goal to <130 mmHg at 1 year. We simultaneously revised our inclusion criteria from SBP ≥120 mmHg, to either SBP ≥130 mmHg, or SBP between 120 and 130 mmHg and a history of hypertension or on antihypertensive medications prior to the stroke or TIA event.

Power analyses were conducted with comparison of the primary outcome (SBP) between the intervention and the control arm. Based on availability of our patients and our capacity, we plan to enroll 516 participants. Using intra-class correlation of the five sites at 0.0085 level, standard deviation of 20 mmHg, three repeated measurements (at baseline, 3 and 12 months), type I error of 0.05, type II error of 0.2 (or equivalent to power of 80%), 2.4 average data points for each subject (corresponding to 30% attrition), and an auto-correlation of 0.2, the effective sample size for the entire study will be 261 (after adjusting for clustering effect of the five recruitment sites). The effect size for SBP as small as 0.25 in standard deviation units or 5.06 mmHg can be considered clinically meaningful in the presence of both moderators and mediators.

With a planned enrollment of 516 and conservative attrition estimates, SUCCEED is powered to detect a clinically meaningful difference in BP between intervention and usual care arms in the presence of moderator and mediator effects. For potential moderators (e.g., age, sex, stroke type and severity, education, country of birth/primary language), we will test power of moderation using f^2^, the most common measure of effect size [43]. To analyze power of potential mediators (e.g., self-efficacy, self-management, social isolation, depression, perceived risk of stroke), we will use indirect effect.

### Recruitment

We recruit participants from both the inpatient and outpatient settings. The research team presents information about SUCCEED to clinicians in the Neurology, Internal Medicine, Family Medicine, Emergency Medicine, and Physical Medicine and Rehabilitation and Therapy departments. We post study flyers in the wards, outpatient clinics, and housestaff offices of these departments. In the inpatient setting, we discuss the study with the inpatient and consult teams taking care of patients with stroke or TIA. In the outpatient setting, we discuss the study with the physicians, clinic coordinators, nurses, and medical assistants.

A potential participant’s clinician obtains verbal permission from the patient before passing his/her name to a member of the research team. In the inpatient setting, the RA either attends inpatient rounds (fostering awareness about the study and prompting the study team during rounds to ask patients for permission for the RA to meet the patient later) or confers with the team after morning rounds to review eligible participants. In the outpatient setting, we use several methods to identify participants: (1) review clinic patient lists for potentially eligible participants; (2) attend clinic and reviewing the list with the clinician, and (3) remind the clinicians to call the RA for potentially eligible patients. Since outpatients may need to leave the clinic immediately after the visit with the clinician, the RA may not be able to speak to an outpatient before he or she leaves, but can call the patient later. Alternatively, potential subjects can also directly call the RA to learn more about the RCT, using the number listed on flyers.

## Methods: assignment of interventions

### Allocation: sequence generation, allocation concealment mechanism, implementation

Before the RCT began, we used computer-assisted stratified randomization of block size of four to develop randomization schedules. The three stratification variables are site, spoken language (English, Spanish, or other [Mandarin/Cantonese and Korean]), and type of stroke (ischemic vs. hemorrhagic). The schedules have an allocation ratio of 1:1 of control and intervention. We use permuted block randomization that stratifies by site to promote periodic balance through the trial and group balance at the end of the trial. The study programmer/analyst generated 28 lists, one for each stratum; we subsequently programmed these lists into the electronic database, Research Electronic Data Capture (REDCap™), for automated randomization. Each row of the list includes a unique subject ID and arm assignment of the study.

The randomization program in REDCap™ is only accessible by the Project Manager, Principal Investigators and Data Manager, and only the Project Manager enters new data to obtain a randomization assignment. For individuals who consent to the study and meet inclusion criteria, the RA assigns a unique study ID and collects baseline data before randomization. After an eligible participant has consented and completed the baseline survey, the RA informs the Project Manager, who enters the appropriate variables to obtain a randomization assignment. The Project Manager notifies the intervention team of participants randomized to the intervention. If possible, the intervention team meets participants upon enrollment; otherwise, the team calls participants to introduce themselves and the study.

### Screening and consent procedure

When a clinician notifies the RA of a potential participant, the RA arranges a face-to-face meeting to determine whether the patient meets eligibility criteria for enrollment. The RA reads to the patient an institutional review board (IRB)-approved script that further describes the SUCCEED trial. If a potential participant directly contacts the RA, the RA reads the IRB-approved script and if the potential participant is interested, a face-to-face meeting will be scheduled. The RAs are bilingual in English and Spanish, and in the case that a subject speaks a language other than English or Spanish, the research team uses volunteers fluent in the appropriate language to translate for the RA. All consent materials were translated into Spanish by an American Translators Association-certified translator. Consent forms were translated into Korean and Chinese with a three-step process using Transperfect® [44]. If the potential participant is interested in participating in the study, the RA (1) asks questions about age and time of stroke onset or TIA occurrence to confirm eligibility; (2) asks the participant questions to determine comprehension of study participation; and (3) measures BP. For those found to be ineligible, the reasons for ineligibility are recorded. If eligible participants choose not to consent to the study, the RA records the reason. Further, the RA asks permission to use the demographic information of those who decline study participation (without obtaining any personally identifiable information) to generate enrollment propensity weights. Enrollment propensity weights will be used to analyze whether characteristics of those eligible for the trial but who declined to participate may impact generalizability of study outcomes.

### Blinding

The RA collects the baseline survey and examination data prior to randomization. Only the Project Manager randomizes participants, and all data related to participants’ activities with the intervention are managed in a separate database that cannot be accessed by the RAs. RAs remain blinded to group assignment throughout the follow-up. The RAs work in offices that are geographically separate from the intervention team to avoid unblinding. If a participant informs the RA at a subsequent evaluation that they are in either arm, a different RA is assigned to perform the evaluation, without discussion regarding the reason.

## Methods: data collection, management, and analysis

### Data collection methods

All RAs are trained using a manual developed by study staff to promote fidelity across sites. The manual instructs in use of REDCap™, recruitment and telephone scripts, eligibility criteria, surveys, study instruments, measurement procedures, and general study office organization. A co-investigator with expertise in biomarker collection developed the sections regarding the collection of BP, anthropometric measurements, and fingerstick blood sample collection with the CardioChek® device and the DBS. Each RA was certified in two phases: (1) after completion of a 2-day training course which included observation; and (2) after successful observation of a collection at the study site. Anthropometric measurements, BP measurements and blood sample collections are periodically reviewed by this co-investigator with biomarker collection expertise to ensure consistency and fidelity, via reports of collected data and in-person observations.

For each measure, we assessed the appropriateness for our patient population, including considerations such as literacy and cultural appropriateness. We used the National Institutes of Neurological Disorders and Stroke (NINDS) Common Data Elements (CDE) if they were available and appropriate [45]. If survey instruments were not CDEs, we tested them with a pilot group of 24 volunteer participants who fit the study’s eligibility criteria. This group provided feedback on the clarity of the questions, evaluation duration, fingerstick DBS and CardioChek®, anthropometric measurements, and survey questions. We performed cognitive testing of the survey measures. We made revisions to the overall instrument for clarity, order, and length. An additional 10 participants provided feedback for the Korean and Chinese surveys.

### Participant retention

We use multiple strategies to promote participant retention and follow-up for the 3-, 8-, and 12-month evaluations. First, we arrange appointments according to the participants’ availability, and include the possibility of evening and weekend visits. Additionally, we attempt to schedule evaluations around participants’ usual care appointments at the healthcare facility. Participants are given the option to have data collected at other study sites, if more convenient. We offer to pay for transportation for those without access to transportation. For individuals unable to return to the healthcare facilities, we offer telephone interviews and data collection at a home visit. Participants receive cash payment at completion of each of the evaluation time points ranging from $10 to $40.

### Data management

Each of the five sites maintains a linking file that includes identifying information (name, address, phone number) and the subject ID. These files are for the purpose of subject tracking, and subject payment tracking. These files are password-protected on a designated computer in a locked office and only the site’s Principal Investigator and research staff have access to the tracking file. Electronic files are kept until the completion of all data collection and the successful creation of the final analytic dataset.

Once data are entered into REDCap™, these data are only retrievable by the Data Management team at UCLA. The Data Management team analyzes these data, which are stored on a UCLA server. No direct identifiers (only study IDs) are kept with these data.

### Statistical methods

We will compare baseline characteristics between the SUCCEED intervention and usual care groups. We will compare continuous variable means using the *t*-test and ordinal or non-Gaussian continuous variables using the Wilcoxon rank sum test. We will compare unordered categorical variables using Chi-square test or Fisher’s exact test.

Enrollment weights based on a logistic regression model will be calculated using demographic data collected from eligible non-participants. If needed, attrition weights will be determined from logistic regression models using demographic data on participants who drop out of the study. We will combine these two weights to form an overall weight (using the inverse of the product of the probabilities of participation). Both raw and adjusted rates using the overall weight will be compared between SUCCEED and usual care arms.

We will conduct intention-to-treat analyses on all directly measured primary and secondary clinical outcomes using ordinal logistic or multiple linear regression models, incorporating the overall weights. Intervention status will be an independent variable in all models. We will compare study outcomes between the control and intervention groups before and after adjusting for potential covariates associated with the outcome measure.

We will use modified Monte Carlo simulation methods to model outcomes related to cost, cost effectiveness, and global risk reduction

There may be underlying intra-site correlations of outcome measures in the collected data, which will potentially impact statistical significance tests of parameter estimates in the analyses. To account for this in our analyses and modeling, robust standard errors will be calculated using Huber-White Sandwich method. Although we will assess for the impact of clustering data structure, we do not expect a large intra-site correlation because four of the five sites are part of the same county healthcare system and we are not aware of differences of outcomes by site. Furthermore, we will include independent dummy variables for each study site to evaluate possible site-level variation in treatment effects.

We will use statistical tests (such as the Shapiro-Wilk test) and graphical analyses (e.g., histograms and box plots) to assess normality of the measures. Based on the nature of distributions, either parametric (e.g., *t*-test and ANOVA for means) or nonparametric tests (Kruskal-Wallis test for medians) will be used. The approaches will include univariate, bivariate, and multivariate modeling.

We will test moderator and mediator effects separately. To assess the moderating effects, we will compare regression models including interaction terms between the independent variables and each possible moderator, and the models that do not include the corresponding interaction terms, using the likelihood ratio test.

We will use the Sobel test [46] and bootstrapping to test mediating effects. We will use the Sobel test to generate a t statistic, which compares the magnitude of the indirect effect to the standard error of measurement. We will compare the t statistic to the normal distribution to determine its significance. Bootstrapping [47] methods will be used to generate a sampling distribution to assess indirect effects of mediators. Baron and Kenny [48] methods will be used to assess the mediating effects through a series of manual regressions. We will use macros from SPSS and SAS that provide output simultaneously for the 3 methods [47, 49, 50]. We will use structural equation modeling to accommodate mediating or moderating effects at the same time.

To check the fidelity (uptake) of the SUCCEED intervention, we will also analyze attendance at SUCCEED clinics, home visits, and the number of telephone coordination of care calls made during enrollment. To evaluate the implementation of the SUCCEED intervention, observations and interviews will be conducted with the intervention staff. Observations of the first APC clinic visit and CHW home visits will clarify the procedures that occur at each clinical study site. Interviews with APC, CHW, and site PIs will further elucidate the intervention processes; barriers and challenges to implementing the intervention; and key intervention components. The observations and interviews will be compared across study sites and between staff to determine the optimal processes for implementation of the intervention.

SUCCEED is a multi-component intervention. It is infeasible to randomize all permutations of these components; thus, our approach to acquiring knowledge of contributions of these different components is to assess, using both qualitative and quantitative methods: (1) extent of implementation of different intervention components, (2) associations of intensity of use or uptake of different intervention components with mediators and with risk factor control outcomes among intervention participants, and (3) perceptions of staff (via interviews with SUCCEED care managers and CHWs) to identify themes about intervention components judged key to effectiveness. Variables will include, for example, number and type of interactions between patients and different care team providers, interactions among care team members, distributions of patient participation in self-management groups, and so forth.

## Cost analysis

To estimate intervention costs, we will take into account equipment costs (e.g. tablets, home BP monitors) and staff time (ACP, CHW, physician, mobile health programmers), including time for intervention development and time spent in training the various providers. We will collect data on intervention costs through interviews and reviews of expense reports for the project. In our analysis of intervention costs, we will distinguish between start-up costs of implementing the new care model, the fixed annual maintenance costs of sustaining the model, and the marginal cost of adding another patient to the model.

We will calculate health care utilization costs from the perspective of LAC-DHS and from a more general perspective applicable nationwide. For the LAC-DHS analysis, we will query the county administrative database to determine the total number of hospitalization days (bed-days), Intensive Care Unit days, Emergency Department visits, outpatient primary care visits, outpatient subspecialty visits related to stroke prevention, stroke prevention-related prescription medications, and laboratory and imaging tests related to stroke prevention. We will obtain the cost equivalents of each of these services from LAC-DHS. For the national analysis, we will obtain unit costs for each health care service from reimbursement rates provided in Medicare fee schedules.

There are several limitations to administrative database utilization data in LAC-DHS. First, Los Angeles County is geographically large; therefore, participants may seek care from non-LAC-DHS facilities. Second, participants without insurance may not qualify for non-emergency insurance coverage; therefore, they may not receive care within LAC-DHS. Third, prior to 2014, LAC-DHS did not have a single medical record number for each individual and the utilization data was incomplete. Since 2014, however, LAC-DHS has developed robust electronic databases to track utilization, and as of March 2015, all facilities are on a single electronic medical record (Cerner) and each individual has a single patient identifier across the system. To address these limitations, we will use survey methods to obtain participants’ healthcare utilization at baseline, 3, 8, 12 months. We have successfully used surveys for tracking healthcare utilization in previous projects [51, 52]. Although surveys are subject to recall bias, randomization will balance this bias across intervention and control groups.

Another important reason for collecting survey data is to obtain information regarding time spent by family members and friends providing informal assistance to the participant. This is particularly important for individuals with stroke, where much of the long-term cost associated with stroke may be related to informal caregiving [53]. The time of informal caregivers will be valued using national average wage rates in the appropriate age-sex cells as recommended by the Panel on Cost-Effectiveness in Health and Medicine [54].

Once we obtain cost data from administrative and survey sources, we will compare total costs for the intervention group (intervention costs plus health services utilization costs) with costs for the control group. If the intervention improves health and saves costs when compared to the control group, the cost analysis will terminate at this point. If the SUCCEED intervention improves health but the costs of the intervention are higher than in the control group, we will conduct a cost-effectiveness analysis. Our primary estimate of within-trial intervention effectiveness, the reduction in SBP, will be derived directly from SUCCEED. This will allow us to compute the cost of the intervention per mm Hg reduction in SBP, an approach used previously [55], which can also be calculated as the cost per clinically meaningful reduction in BP. This method requires minimal assumptions or modeling, since it uses data collected from participants in the trial. Estimating cost-effectiveness based on within-trial data on BP lowering is limited by two factors – 1) inability to compare the cost-effectiveness of this intervention with other interventions not focused on BP and 2) not accounting for long-term costs and benefits beyond the trial. To improve comparability of SUCCEED with other interventions, we will measure effectiveness using quality adjusted life years (QALYs), [54] combining information on health related quality of life (HRQOL) and mortality. To calculate the HRQOL of the intervention and control patients, we will survey participants using the SF-6D [56]. We will obtain vital status of individuals throughout the study for mortality calculations.

In order to demonstrate the clinical relevance of the reduction of multiple stroke risk factors we will estimate the combined impact of SUCCEED on cardiovascular risk at 3 years. As noted above, the “global risk reduction” outcome estimates the relative reduction in risk for recurrent stroke, and for fatal and nonfatal cardiovascular disease (stroke or myocardial infarction) at 12 months. We will modify the relative global risk reduction calculation to estimate risk for stroke and CVD at 36 months, using an alternative ratio of stroke: CVD in order to account for the rapid decline in risk of recurrent stroke relative to risk of myocardial infarction following a stroke event [57]. Two sources of uncertainty affect the precision of our estimate of the reduction in risk of fatal and nonfatal stroke and CVD. We will use bootstrapping techniques to account for uncertainty in our data (observed changed in risk factors); and we will use Monte Carlo simulation methods to account for uncertainty in the effectiveness of each component intervention. Additional details on the bootstrapping-simulation methods will be available in a subsequent global risk reduction manuscript.

## Methods: monitoring

### Data monitoring

For Care Management studies such as SUCCEED, a Data Safety Monitoring Board is not usually required. The APCs and CHWs monitor vascular risk factor control and resource utilization of subjects randomized to the intervention. Through telephone calls, clinic visits, home visits, and CDSMP workshops, they ask subjects about the steps they are taking to lower the risk of stroke.

### Harms

Adverse experiences are reported as required by the IRB at each site. There are no additional US Food and Drug Administration regulations because we are not studying a biological agent. The Manual of Operations includes detailed definitions of adverse experiences, a table for grading their severity, and details of how clinical sites are to report them.

### Auditing

A co-investigator with expertise in biomarker collection periodically conducts site visits for quality assurance regarding biomarker collection by the RAs and reviews data on protocol fidelity periodically downloaded by the study programmer from REDcap™. Additionally, the Project Manager reviews the data entered by the RAs and locks the forms monthly.

## Sensitivity analyses

Moderator analyses described above provide estimates of possible effect modification among pre-specified subgroups in Table 1. Exploring possible variation in outcomes among sociodemographic subgroups facilitates equity analyses to determine whether the intervention preferentially benefits socially disadvantaged individuals. Additional sensitivity analyses will be performed to examine how various assumptions related to the efficacy and cost of individual components impact simulated estimates of the overall impact of the intervention on clinical global risk reduction and cost-effectiveness.

## Discussion

The optimal BP goal after stroke remains unclear in the absence of RCTs designed to answer the question. The goal, therefore, may change as new RCT findings become available. Nevertheless, the SUCCEED intervention is designed to address all major cardiovascular risk factors, in addition to BP. Although a composite cardiovascular risk score would have been a reasonable alternative to BP as a primary outcome, existing composite scores were derived from populations free of stroke. We have developed and will use this trial to test a composite tool for assessing relative risk of recurrent stroke [58]. Second, since this is a pragmatic trial, the clinical settings vary between sites and we have allowed for heterogeneity; however, we have strategies in place to heighten fidelity in the core aspects of the intervention. In addition, there may be site-specific differences in usual care and temporal changes in usual care. By testing this intervention in real world settings, we will maximize external validity. Third, it will be difficult to assess which components of the intervention explain the effectiveness (or lack thereof); however, we will employ post-hoc subgroup analyses and assess the effects of mediators and moderators. Fourth, contamination of the usual care arm is possible. However, the only providers who potentially care for participants in both arms of the trial are the site PIs; they do not share intervention tools or educational materials with participants. The APCs and CHWs do not interact with usual care participants.

Individuals with stroke or TIA are often high-cost, high-needs patients; therefore, if this coordinated care management intervention is effective, it can have far-reaching consequences. By using an interdisciplinary team, we have drawn upon various skillsets in a cost-effective manner, and anticipate improving the quality and effectiveness of care. In addition, we anticipate improving efficiency and care quality by using evidence-based protocols embedded into a mobile electronic platform that offers real-time decision support, an avenue for communication, and a tool for panel management. By including community representatives and stroke survivors in the development of the intervention, and employing CHWs from the communities that are served, we have culturally tailored the intervention and are better equipped to address potential barriers, understand participants’ needs, and provide patient-centered care.

Although this intervention is designed to address disparities in care and outcomes in vulnerable, socioeconomically disadvantaged populations, it may be applicable to a variety of healthcare settings. Furthermore, since individuals with stroke and TIA have multiple cardiovascular comorbidities, many of the tools we developed may be applicable to a large proportion of high-cost, high-needs patients at high risk for CVD. Finally, the care management model developed in this trial is applicable to most chronic conditions.

Once the RCT is completed, the materials and products will be available to the research, clinical and public health communities. Dimagi’s software is open source.

## Progress to date

We have enrolled 357 of the 516 participants (Table 3). The mean age is 57.2 years (SD 8.7). The majority are men (64.5%). With respect to race, 66.2% are White, 17.7% Black, 7.3% Asian, 2.5% Native American or Alaskan Native, and 3.7% >1 race. The majority (70.9%) are Hispanic. Most participants are born outside of the United States (72.7%) and 61.9% have less than a high school education. Approximately half (52.5%) were working prior to the stroke. The majority of events were ischemic strokes or TIAs (83.2%) and the remainder were intracerebral hemorrhages. The mean systolic BP at baseline was 145.5 mmHg. Three quarters of the population was either overweight or obese. Most had mild to moderate strokes (NIH SS <15). Nearly half of the participants had moderately severe or severe disability at enrollment. Nearly half the participants had a history of smoking; 24.1% smoked in the year prior to the stroke. With respect to medical comorbidities, 23% had a prior history of stroke, 51.7% had dyslipidemia and 48.3% had diabetes by self report.

**Table 3:** Sociodemographic and clinical characteristics of participants enrolled to date

	Eligible enrolled (*N* = 357)
Sociodemographic Characteristics	
Age, years, mean (SD)	57.2 (8.7)
Male, *n* (%)	229 (64.5)
Race, *n* (%)	
White	235 (66.2)
Black	63 (17.7)
Asian	26 (7.3)
American Indian/Alaskan Native	9 (2.5)
Native Hawaiian/Other Pacific Islander	3 (0.8)
More than one race	13 (3.7)
Unknown	6 (1.7)
Ethnicity: Hispanic, *n* (%)	251 (70.9)
Born in the United States, *n* (%)	97 (27.3)
Living with at least one other adult, *n* (%)	314 (88.5)
Education, *n* (%)	
Some college	107 (30.4)
Associate degree: academic, occupational, technical or vocational program	1 (0.3)
At least high school graduate or equivalent	26 (7.4)
Some high school	81 (23.0)
8^th^ grade or less	137 (38.9)
Working for pay, part- or full-time, prior to stroke, *n* (%)	189 (53.5)
Clinical Characteristics	
Stroke Type, *n* (%)	
Ischemic / TIA	297 (83.2)
Intracerebal hemorrhage	60 (16.8)
Systolic blood pressure, mm Hg, mean (SD)	145.4 (18.4)
BMI category, *n* (%)	
Underweight (<18 kg/m^2^)	1 (0.7)
Normal (18–24.9 kg/m^2^)	37 (24.7)
Overweight (25–29.9 kg/m^2^)	61 (40.7)
Obese (≥30 kg/m^2^)	51 (34.0)
NIH stroke score, *n* (%)	
Mild (1–5)	225 (63.4)
Moderate (6–14)	121 (34.1)
Severe (15–24)	9 (2.5)
Very severe (≥25)	0
Modified Rankin Scale, *n* (%)	
No disability	25 (7.0)
No significant disability	60 (16.9)
Slight disability	60 (16.9)
Moderate disability	53 (14.9)
Moderately severe disability	112 (31.5)
Severe disability	45 (12.7)
History of smoking, *n* (%)	160 (45.3)
Smoked in the year prior to the stroke, *n* (%)	85 (24.1)
Medical History, *n* (%)	
Prior stroke	80 (23.0)
Heart attack	32 (9.0)
Atrial fibrillation	25 (7.1)
Congestive heart failure	26 (7.4)
Dyslipidemia	182 (51.7)
Cancer	14 (4.0)
Diabetes	171 (48.3)

## Abbreviations

APC: Advanced Practice Clinician
BMI: Body mass index
BP: Blood pressure
CCM: Chronic care model
CDE: Common data elements
CDMP: Chronic Disease Self-Management Program
CHW: Community Health Worker
CRP: C reactive protein
DBS: Dried blood spot
HbA1c: Hemoglobin A1c
HCWDP: Healthcare Workforce Development Program
HDL: High density lipoprotein
HIPAA: Health Insurance Portability and Accountability Act
ICC: Intraclass correlation
IRB: Institutional Review Board
LAC-DHS: Los Angeles County Department of Health Services
LDL: Low density lipoprotein
LRT: Likelihood ratio test
NINDS: National Institute of Neurological Disorders and Stroke
NP: Nurse Practitioner
PA: Physician Assistant
PHQ: Patient health questionnaire
RA: Research Assistant
RCT: Randomized controlled trial
REDCap™: Research electronic data capture
SAS: Statistical analysis system
SBP: Systolic blood pressure
SPSS: Statistical Package for the Social Sciences
SUCCEED: Secondary stroke prevention by Uniting Community and Chronic care model teams Early to End Disparities
SUSTAIN: Systemic Use of STroke Averting INterventions
TIA: Transient ischemic attack
WERC: Worker Education and Resource Center
WLCAC: Watts Labor Community Action Committee

## References

[1] Bodenheimer T, Berry-Millett R (2009). Follow the money—controlling expenditures by improving care for patients needing costly services. N Engl J Med.

[2] Bodenheimer TS, Smith MD (2013). Primary care: proposed solutions to the physician shortage without training more physicians. Health Aff (Millwood).

[3] Reuben DB (2011). Physicians in supporting roles in chronic disease care: the CareMore model. J Am Geriatr Soc.

[4] Milstein A, Gilbertson E (2009). American medical home runs. Health Aff (Millwood).

[5] Bell JF, Krupski A, Joesch JM, West II, Atkins DC, Court B, Mancuso D, Roy-Byrne P (2015). A randomized controlled trial of intensive care management for disabled Medicaid beneficiaries with high health care costs. Health Serv Res.

[6] Mozaffarian D, Benjamin EJ, Go AS, Arnett DK, Blaha MJ, Cushman M, Das SR, de Ferranti S, Despres JP, Fullerton HJ (2016). Heart disease and stroke statistics-2016 update: a report from the American heart association. Circulation.

[7] Egan BM, Zhao Y, Axon RN (2010). US trends in prevalence, awareness, treatment, and control of hypertension, 1988–2008. JAMA.

[8] Furie KL, Kasner SE, Adams RJ, Albers GW, Bush RL, Fagan SC, Halperin JL, Johnston SC, Katzan I, Kernan WN (2011). Guidelines for the prevention of stroke in patients with stroke or transient ischemic attack: a guideline for healthcare professionals from the American heart association/American stroke association. Stroke.

[9] O’Donnell MJ, Xavier D, Liu L, Zhang H, Chin SL, Rao-Melacini P, Rangarajan S, Islam S, Pais P, Mcqueen MJ (2010). Risk factors for ischaemic and intracerebral haemorrhagic stroke in 22 countries (the INTERSTROKE study): a case-control study. Lancet.

[10] Whitworth JA (2003). 2003 World Health Organization (WHO)/International Society of Hypertension (ISH) statement on management of hypertension. J Hypertens.

[11] Cheng EM, Jolly D, Jones LA, Cohen SN (2005). Modest improvement in risk factor control after admission for a stroke or transient ischemic attack. J Stroke Cerebrovasc Dis.

[12] Razmara A, Ovbiagele B, Markovic D, Towfighi A. Patterns and predictors of blood pressure treatment, control, and outcomes among stroke survivors in the United States. J Stroke Cerebrovasc Dis. 2016;25(4):857–65. 10.1016/j.jstrokecerebrovasdis.2015.12.027 26778599

[13] Lin MP, Ovbiagele B, Markovic D, Towfighi A: “Life’s simple 7” and long-term mortality after stroke. *J Am Heart Assoc* 2015, 4 (11). 10.1161/JAHA.114.001470 PMC4845235 26588943

[14] McManus M, Ovbiagele B, Markovic D, Towfighi A (2015). Association of Insurance Status with Stroke-Related Mortality and Long-term Survival after Stroke. J Stroke Cerebrovasc Dis.

[15] Cruz-Flores S, Rabinstein A, Biller J, Elkind MS, Griffith P, Gorelick PB, Howard G, Leira EC, Morgenstern LB, Ovbiagele B (2011). Racial-ethnic disparities in stroke care: the American experience: a statement for healthcare professionals from the American Heart Association/American Stroke Association. Stroke.

[16] Bodenheimer T, Wagner EH, Grumbach K (2002). Improving primary care for patients with chronic illness. JAMA.

[17] Bodenheimer T, Wagner EH, Grumbach K (2002). Improving primary care for patients with chronic illness: the chronic care model, Part 2. JAMA.

[18] Cheng EM, Cunningham WE, Towfighi A, Sanossian N, Bryg RJ, Anderson TL, Guterman JJ, Gross-Schulman SG, Beanes S, Jones AS (2011). Randomized, controlled trial of an intervention to enable stroke survivors throughout the Los Angeles County safety net to “stay with the guidelines”. Circ Cardiovasc Qual Outcomes.

[19] Brownstein JN, Chowdhury FM, Norris SL, Horsley T, Jack L, Zhang X, Satterfield D (2007). Effectiveness of community health workers in the care of people with hypertension. Am J Prev Med.

[20] Gary TL, Batts-Turner M, Yeh HC, Hill-Briggs F, Bone LR, Wang NY, Levine DM, Powe NR, Saudek CD, Hill MN (2009). The effects of a nurse case manager and a community health worker team on diabetic control, emergency department visits, and hospitalizations among urban African Americans with type 2 diabetes mellitus: a randomized controlled trial. Arch Intern Med.

[21] Allen JK, Dennison-Himmelfarb CR, Szanton SL, Bone L, Hill MN, Levine DM, West M, Barlow A, Lewis-Boyer L, Donnelly-Strozzo M (2011). Community Outreach and Cardiovascular Health (COACH) Trial: a randomized, controlled trial of nurse practitioner/community health worker cardiovascular disease risk reduction in urban community health centers. Circ Cardiovasc Qual Outcomes.

[22] Hill MN, Han HR, Dennison CR, Kim MT, Roary MC, Blumenthal RS, Bone LR, Levine DM, Post WS (2003). Hypertension care and control in underserved urban African American men: behavioral and physiologic outcomes at 36 months. Am J Hypertens.

[23] Fedder DO, Chang RJ, Curry S, Nichols G (2003). The effectiveness of a community health worker outreach program on healthcare utilization of west Baltimore City Medicaid patients with diabetes, with or without hypertension. Ethn Dis.

[24] Babamoto KS, Sey KA, Camilleri AJ, Karlan VJ, Catalasan J, Morisky DE (2009). Improving diabetes care and health measures among hispanics using community health workers: results from a randomized controlled trial. Health Educ Behav.

[25] Dennison CR, Post WS, Kim MT, Bone LR, Cohen D, Blumenthal RS, Rame JE, Roary MC, Levine DM, Hill MN (2007). Underserved urban african american men: hypertension trial outcomes and mortality during 5 years. Am J Hypertens.

[26] Philis-Tsimikas A, Fortmann A, Lleva-Ocana L, Walker C, Gallo LC (2011). Peer-led diabetes education programs in high-risk Mexican Americans improve glycemic control compared with standard approaches: a Project Dulce promotora randomized trial. Diabetes Care.

[27] Norris SL, Chowdhury FM, Van Le K, Horsley T, Brownstein JN, Zhang X, Jack L, Satterfield DW (2006). Effectiveness of community health workers in the care of persons with diabetes. Diabet Med.

[28] Dromerick AW, Gibbons MC, Edwards DF, Farr DE, Giannetti ML, Sanchez B, Shara NM, Fokar A, Jayam-Trouth A, Ovbiagele B (2011). Preventing recurrence of thromboembolic events through coordinated treatment in the district of Columbia. Int J Stroke.

[29] Egan M, Anderson S, Mctaggart J (2010). Community navigation for stroke survivors and their care partners: description and evaluation. Top Stroke Rehabil.

[30] Towfighi A, Gallup M, Tai W, Guevara E, Ovbiagele B. Differences in index ischemic stroke characteristics in a multiethnic medically underserved population. International Stroke Conference, San Antonio; 2010.

[31] Ramirez M, Wu S, Towfighi A, Wacksman J, Sivers-Teixeira T, Haber H, Vickrey BG. International Symposium on Human Factors and Ergonomics in Health Care. Using mobile health tools to support team-based approaches for chronic disease care. San Diego; 2016.

[32] Rashid P, Leonardi-Bee J, Bath P (2003). Blood pressure reduction and secondary prevention of stroke and other vascular events: a systematic review. Stroke.

[33] Turnbull F, Blood Pressure Lowering Treatment Trialists C (2003). Effects of different blood-pressure-lowering regimens on major cardiovascular events: results of prospectively-designed overviews of randomised trials. Lancet.

[34] Lawes CM, Bennett DA, Feigin VL, Rodgers A (2004). Blood pressure and stroke: an overview of published reviews. Stroke.

[35] Dorresteijn JA, van der Graaf Y, Spiering W, Grobbee DE, Bots ML, Visseren FL, Secondary Manifestations of Arterial Disease Study G (2012). Relation between blood pressure and vascular events and mortality in patients with manifest vascular disease: J-curve revisited. Hypertension.

[36] Cooper-Dehoff RM, Gong Y, Handberg EM, Bavry AA, Denardo SJ, Bakris GL, Pepine CJ (2010). Tight blood pressure control and cardiovascular outcomes among hypertensive patients with diabetes and coronary artery disease. JAMA.

[37] Cushman WC, Evans GW, Byington RP, Goff DC, Grimm RH, Cutler JA, Simons-Morton DG, Basile JN, Corson MA, Accord Study Group (2010). Effects of intensive blood-pressure control in type 2 diabetes mellitus. N Engl J Med.

[38] Vamos EP, Harris M, Millett C, Pape UJ, Khunti K, Curcin V, Molokhia M, Majeed A (2012). Association of systolic and diastolic blood pressure and all cause mortality in people with newly diagnosed type 2 diabetes: retrospective cohort study. BMJ.

[39] Boan AD, Lackland DT, Ovbiagele B (2014). Lowering of blood pressure for recurrent stroke prevention. Stroke.

[40] Ovbiagele B (2013). Low-normal systolic blood pressure and secondary stroke risk. J Stroke Cerebrovasc Dis.

[41] Ovbiagele B, Diener HC, Yusuf S, Martin RH, Cotton D, Vinisko R, Donnan GA, Bath PM, Investigators P (2011). Level of systolic blood pressure within the normal range and risk of recurrent stroke. JAMA.

[42] Lin MP, Ovbiagele B, Markovic D, Towfighi A (2015). Systolic blood pressure and mortality after stroke: too low, no go?. Stroke.

[43] Aiken LS, West SG (1991). Multiple regression: testing and interpreting interactions.

[44] TransPerfect Translations International. 3 Park Avenue, 39th Floor New York, New York 10016.

[45] Grinnon ST, Miller K, Marler JR, Lu Y, Stout A, Odenkirchen J, Kunitz S (2012). National Institute of Neurological Disorders and Stroke Common Data Element Project—approach and methods. Clin Trials.

[46] Sobel Mel S (1982). Asymptotic confidence intervals for indirect effects in structural equation models. Sociol Methodol.

[47] Shrout PE, Bolger N (2002). Mediation in experimental and nonexperimental studies: new procedures and recommendations. Psychol Methods.

[48] Baron RM, Kenny DA (1986). The moderator-mediator variable distinction in social psychological research: conceptual, strategic, and statistical considerations. J Pers Soc Psychol.

[49] Paffenbarger RS, Wing AL, Hyde RT (1995). Physical activity as an index of heart attack risk in college alumni. 1978. Am J Epidemiol.

[50] Preacher KJ, Hayes AF (2008). Asymptotic and resampling strategies for assessing and comparing indirect effects in multiple mediator models. Behav Res Methods.

[51] Mangione CM, Brown A, Sarkisian C, Brusuelas RJ, Norris K, Steers N, Davison M, Ettner S, Ganz D, Funnell MM (2007). Randomized community-based intervention to improve self-management of diabetes among older African Americans and Latinos. J Gen Intern Med.

[52] Duru OK, Ettner SL, Vassar SD, Chodosh J, Vickrey BG (2009). Cost evaluation of a coordinated care management intervention for dementia. Am J Manag Care.

[53] Hickenbottom SL, Fendrick AM, Kutcher JS, Kabeto MU, Katz SJ, Langa KM (2002). A national study of the quantity and cost of informal caregiving for the elderly with stroke. Neurology.

[54] Gold MR (1996). Cost-effectiveness in health and medicine.

[55] Logan AG, Milne BJ, Achber C, Campbell WP, Haynes RB (1981). Cost-effectiveness of a worksite hypertension treatment program. Hypertension.

[56] Brazier J, Roberts J, Deverill M (2002). The estimation of a preference-based measure of health from the SF-36. J Health Econ.

[57] Hankey GJ (2014). Secondary stroke prevention. Lancet Neurol.

[58] Richards A, Jackson N, Cheng E, Towfighi A, Bryg R, Brown A, Sanossian N, Barry F, Li N, Vickrey BG. Global risk reduction: a novel method to estimate the impact of multiple intervention trials to reduce stroke disparities. International Stroke Conference, Los Angeles; 2016.

[59] Craig CL, Marshall AL, Sjostrom M, Bauman AE, Booth ML, Ainsworth BE, Pratt M, Ekelund U, Yngve A, Sallis JF (2003). International physical activity questionnaire: 12-country reliability and validity. Med Sci Sports Exerc.

[60] UCLA Center for Health Policy Research. California Health Interview Survey 2011-2012 (CHIS 2011-2012).

[61] Centers for Disease Control and Prevention (2013). Behavioral risk factor surveillance system survey questionnaire.

[62] Meschia JF, Brott TG, Chukwudelunzu FE, Hardy J, Brown RD, Meissner I, Hall LJ, Atkinson EJ, O’Brien PC (2000). Verifying the stroke-free phenotype by structured telephone interview. Stroke.

[63] Marin G, Gamba RJ (1996). A New measurement of acculturation for hispanics: the bidimensional acculturation scale for Hispanics (BAS). Hisp J Behav Sci.

[64] Schneider AT, Pancioli AM, Khoury JC, Rademacher E, Tuchfarber A, Miller R, Woo D, Kissela B, Broderick JP (2003). Trends in community knowledge of the warning signs and risk factors for stroke. JAMA.

[65] Haun J, Luther S, Dodd V, Donaldson P (2012). Measurement variation across health literacy assessments: implications for assessment selection in research and practice. J Health Commun.

[66] Simoni JM, Kurth AE, Pearson CR, Pantalone DW, Merrill JO, Frick PA (2006). Self-report measures of antiretroviral therapy adherence: a review with recommendations for HIV research and clinical management. AIDS Behav.

[67] Chesney MA, Ickovics JR, Chambers DB, Gifford AL, Neidig J, Zwickl B, Wu AW (2000). Self-reported adherence to antiretroviral medications among participants in HIV clinical trials: the AACTG adherence instruments. Patient Care Committee & Adherence Working Group of the Outcomes Committee of the Adult AIDS Clinical Trials Group (AACTG). AIDS Care.

[68] Lorig KR, Sobel DS, Ritter PL, Laurent D, Hobbs M (2001). Effect of a self-management program on patients with chronic disease. Eff Clin Pract.

[69] Luszczynska A, Scholz U, Schwarzer R (2005). The general self-efficacy scale: multicultural validation studies. J Psychol.

[70] Powers BJ, Oddone EZ, Grubber JM, Olsen MK, Bosworth HB (2008). Perceived and actual stroke risk among men with hypertension. J Clin Hypertens (Greenwich).

[71] Wong ST, Nordstokke D, Gregorich S, Perez-Stable EJ (2010). Measurement of social support across women from four ethnic groups: evidence of factorial invariance. J Cross Cult Gerontol.

[72] Radloff L. The CES-D scale: A self report depression scale for research in the general population. Applied Psychological Measurement. 1977;1(3):385–401.

[73] Kharroubi SA, Brazier JE, Roberts J, O’Hagan A (2007). Modelling SF-6D health state preference data using a nonparametric Bayesian method. J Health Econ.

[74] Glasgow RE, Wagner EH, Schaefer J, Mahoney LD, Reid RJ, Greene SM (2005). Development and validation of the Patient Assessment of Chronic Illness Care (PACIC). Med Care.

[75] Harris-Kojetin LD, Fowler FJ, Brown JA, Schnaier JA, Sweeny SF (1999). The use of cognitive testing to develop and evaluate CAHPS 1.0 core survey items. Consumer Assessment of Health Plans Study. Med Care.

